# Transformers in RNA structure prediction: A review

**DOI:** 10.1016/j.csbj.2025.03.021

**Published:** 2025-03-17

**Authors:** Mayank Chaturvedi, Mahmood A. Rashid, Kuldip K. Paliwal

**Affiliations:** Signal Processing Laboratory, School of Engineering and Built Environment, Griffith University, Brisbane, QLD, 4111, Australia

**Keywords:** RNA structure prediction, Deep learning, Self-attention, Transformers

## Abstract

The Transformer is a deep neural network based on the self-attention mechanism, designed to handle sequential data. Given its tremendous advantages in natural language processing, it has gained traction for other applications. As the primary structure of RNA is a sequence of nucleotides, researchers have applied Transformers to predict secondary and tertiary structures from RNA sequences. The number of Transformer-based models in structure prediction tasks is rapidly increasing as they have performed on par or better than other deep learning networks, such as Convolutional and Recurrent Neural Networks. This article thoroughly examines Transformer-based RNA structure prediction models. Through an in-depth analysis of the models, we aim to explain how their architectural innovations improve their performances and what they still lack. As Transformer-based techniques for RNA structure prediction continue to evolve, this review serves as both a record of past achievements and a guide for future avenues.

## Highlights

• Reviews state-of-the-art Transformer-based RNA structure prediction tools.
• Presents a detailed technical analysis of RNA 2D and 3D structure prediction tools.
• Highlights the latest advances in Transformer-based structure prediction methods.
• Identifies the current challenges and future trends in RNA structure prediction.

## Graphical abstract

**Figure:**
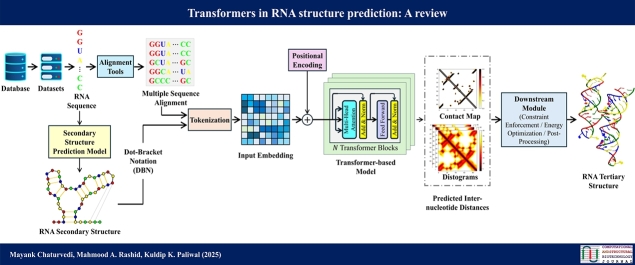

## 1 Introduction

**Fig. 1:**
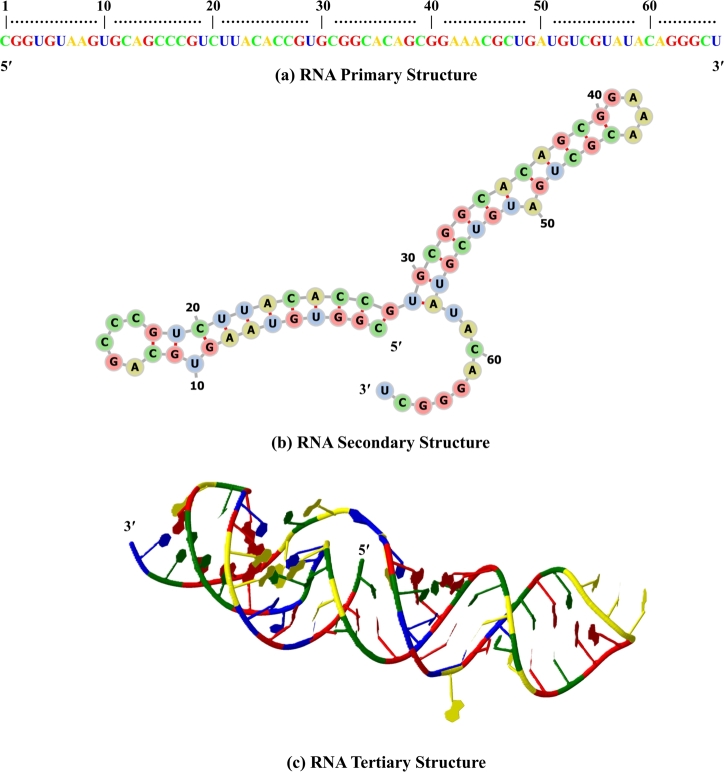
**Representation of the structures of the severe acute respiratory syndrome coronavirus 2 (SARS-CoV-2) frameshifting pseudoknot****[6]****: (a) Primary structure showing the linear sequence of nucleotides, (b) Secondary structure depicting base-pairing interactions, and (c) Tertiary structure showing the three-dimensional folding of the RNA molecule.** The prediction and visualization of the secondary structure was obtained using Forna [15]. The tertiary structure is predicted by a Transformer-based 3D structure prediction model, trRosettaRNA [16], and visualized using Jmol [17].

Ribonucleic acid (RNA) is a fundamental molecule in all living organisms and viruses. RNA is essential for carrying genetic information, protein synthesis, and gene expression [1], [2]. It contains four nucleotides, adenine (A), cytosine (C), guanine (G), and uracil (U). RNA can fold into different structures to meet various functional demands, from simple genetic information encoding to complex catalysis and regulation [3], [4]. RNA structures can be analyzed at three levels: primary, secondary (2D), and tertiary (3D), facilitating a better understanding of their functions [5]. The structures of the severe acute respiratory syndrome coronavirus 2 (SARS-CoV-2) frameshifting pseudoknot [6], [7] are shown in Fig. 1, illustrating the primary, secondary, and tertiary structures. The primary structure of RNA is a linear sequence of nucleotides attached by 5′-3′ phosphodiester bonds between ribose sugars [8]. Single-strand RNAs form secondary structures as a result of the base pairing of complementary nucleotides through hydrogen bonds. The canonical base pairs for RNA are the Watson–Crick base pairs (A–U and G–C) and the wobble base pair (G–U) [9], [10]. Based on the number of closing base pairs, the base pairing can result in a nested structure such as stackings, hairpin loops, internal loops, bulge loops, multi-branch loops, and external loops [11], [12], [13]. The RNA secondary structure folds further into the tertiary structure due to interactions between the elements of the secondary structure or non-canonical base pairing [14].

The RNA structure provides critical information on its biological functions and facilitates applications in biotechnology and medicine [18], [19], [20]. There can be two approaches to determining tertiary or secondary structures from a given RNA sequence or primary structure. The first approach is to measure the structure through wet lab experiments. X-ray crystallography, cryo-electron microscopy (Cryo-EM), and nuclear magnetic resonance (NMR) spectroscopy are the experimental techniques commonly used to determine precise tertiary structures [21]. For a reliable secondary structure, Selective 2′-Hydroxyl Acylation analyzed by Primer Extension (SHAPE) [22], [23] and chemical probing [24], [25], [26], [27] are generally used. However, despite being highly accurate, experimental techniques are time consuming, expensive, and require specialized equipment and expertise [28], [29]. Recently, tools such as RNAProbe [30] and RNAthor [31] have been developed that can potentially reduce the time and expertise required to interpret the results by streamlining the analysis of chemical screening experiments. The second approach to finding the structure is the application of computational methods. In recent years, the increased availability of high-performance computational resources, combined with the rapid growth of experimentally determined RNA sequence databases, has resulted in significant advances in the predictive accuracy of computational techniques. Computational techniques complement experimental methods, and their correct coordination can lead to highly accurate predictions of secondary or tertiary structures.

One of the earliest computational techniques proposed by Nussinov et al. [32] employed dynamic programming to find the RNA secondary structure by maximizing the number of base pairs. Zuker et al. [33] used dynamic programming to introduce an algorithm that finds the most stable RNA secondary structure by calculating the lowest free energy. Subsequently, several methods were developed using thermodynamic parameters [34], [35]. RNAfold [36] and LinearFold [37] calculate the minimum free energy (MFE) to predict the RNA secondary structures. Models such as Mfold [38], Vfold [39], MC-fold [40] and NUPACK [41] use Turner's nearest neighbor model [42] for the prediction of RNA secondary structure. UNAfold [43] and RNAstructure [44] use both the MFE and the nearest neighbor model to predict secondary structures. However, models using thermodynamic parameters struggled with long sequences due to their inability to capture long-range interactions. Comparative sequence analysis [45], [46], [47] and covariation-based approaches [48], [49], [50], [51] improved prediction accuracy by employing multiple sequence alignment (MSA). These methods have been beneficial in identifying conserved structure motifs and inter-RNA secondary structures by comparing RNA sequences with homologous species. Methods that use MSAs as input can improve structure prediction by taking advantage of co-evolution information obtained from MSA [52]. However, training on single query sequences does not depend on the often tedious and complex process of constructing MSAs and offers notable advantages in speed and flexibility [53].

However, since the advent of machine learning (ML), there has been a paradigm shift in this field. ML algorithms, including support vector machines (SVM), conditional log-linear models (CLLM), maximum likelihood estimation, and max-margin frameworks, have been prominently used for RNA structure prediction through methods such as CONTRAfold [11], TORNADO [54], and MXFold [55]. Deep learning (DL) models have made learning the complex patterns of RNA sequence data much simpler and more efficient. Numerous DL models used the Recurrent Neural Network (RNN) architecture to efficiently capture sequential dependencies in RNA sequences [56], [57], [58], [59]. To preserve spatial relationships using convolutional layers and reduce dimensionality using pooling layers, models such as CDPfold [60], SPOT-RNA [61], Ufold [62], and MXfold2 [63] employed the Convolutional Neural Network (CNN) and significantly improved prediction accuracy. The RNNs process the input sequentially, which slows training and prediction [64]. They also struggle to capture long-range dependencies [65]. In contrast, CNNs can perform parallel computations increasing model speed [66], [67], [68]. However, CNNs are also not suitable for capturing long-range dependencies. Models using Graph Neural Network (GNN), including Graph Convolutional Networks [69] and Graph Isomorphism Networks [70], can process graph-structured data efficiently. However, GNN-based models can be computationally intensive for higher layers and require significant computational costs [71], [72].

The prediction models for RNA tertiary structures can be categorized as physics-based, knowledge-based, and ML/DL-based [73]. Physics-based models such as HiRE-RNA [74], iFoldRNA v2 [75], and RNAJP [76] depend on physical principles for tertiary structure prediction. These models demand significant computational resources as they thoroughly investigate folding pathways to determine stable conformations. Knowledge-based tools, including RNAComposer [77], SimRNA [78], FARFAR2 [79], 3dRNA [80], and FebRNA [81], use scoring functions and fragments from known RNA structures to make predictions. ML/DL models such as RNA3DCNN [82], PaxNet [83], and epRNA [84] use ML/DL techniques to predict RNA tertiary structures. Like knowledge-based tools, ML/DL-based techniques use known RNA structures from wet lab experiments to train the models. Several researchers have critically reviewed the ML- and DL-based techniques developed for RNA structure prediction [85], [86], [87], [88], [89], [90], [91], [92].

Recently, a new neural network architecture known as Transformer is gaining momentum, which uses self-attention mechanisms to extract intrinsic features. The Transformer architecture was proposed by Vaswani et al. in 2017 [93] for machine translation. Due to their high efficiency and simple architecture, Transformers are being applied across various domains of artificial intelligence (AI), including computer vision [94], [95], [96], signal processing [97], [98], reinforcement learning [99], [100], [101], [102], and financial fraud detection [103], [104], [105], [106]. The success of the Transformer architecture in various fields motivated researchers to apply it to RNA structure prediction. Consequently, the Transformer architecture is emerging as a strong alternative to established architectures such as CNN and RNN for RNA structure prediction tasks. The exponential growth of Transformer-based models for structure prediction has made it challenging to keep up with all the developments in this field. In this context, reviewing existing work is necessary and beneficial for the community.

This article presents an overview of recent advances in Transformer-based models used for structure prediction, as well as discusses current challenges and future directions. The rest of the article is organized as follows. The architecture and key components of the vanilla Transformer are introduced in Section 2. Section 3 reviews Transformer-based techniques for secondary and tertiary structure prediction. Section 4 discusses existing challenges and future trends. Finally, the key findings are summarized in the last section (Section 5) of the article.

## 2 Overview of transformer architecture

**Fig. 2:**
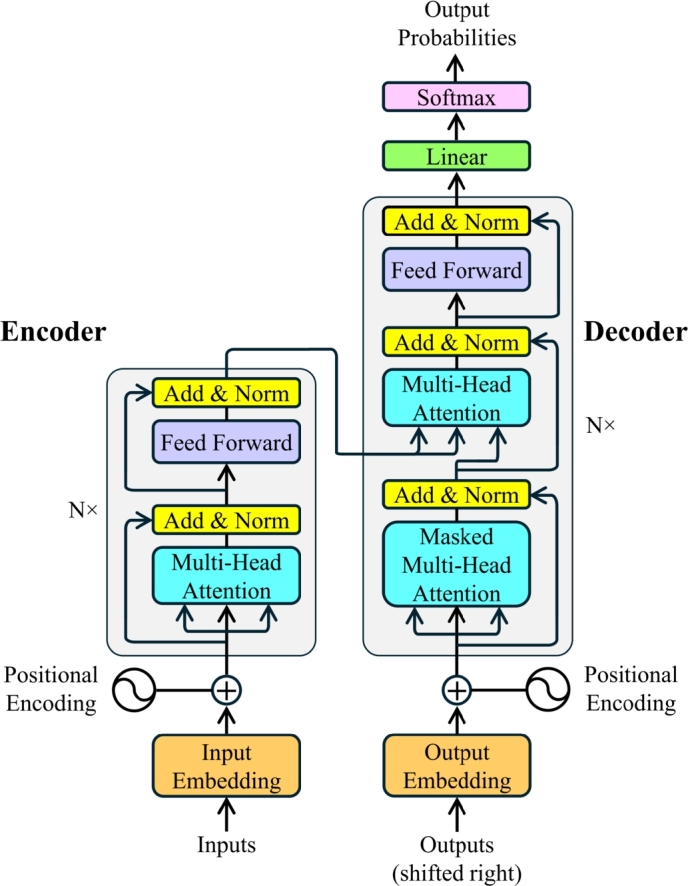
A schematic diagram of the Transformer architecture [93].

Before diving into Transformer-based structure prediction techniques, it is imperative to be familiar with the main components of vanilla Transformer and its functions. The schematic diagram of the Transformer architecture is shown in Fig. 2. The model architecture is divided into two principal components, the encoder and the decoder, comprising several identical blocks. Each block contains multi-head self-attention and point-wise feed-forward neural network sub-layers. The following subsections describe each key element in detail.

### 2.1 Encoder

The encoder of the vanilla Transformer contains a stack of six layers, where each layer incorporates a sub-layer of multi-head self-attention mechanism and a sub-layer of point-wise fully connected feed-forward neural network. Residual connections [107] are applied around the sub-layers, followed by layer normalization [108]. The residual connection facilitates an efficient gradient flow through the network layers, and the layer normalization maintains consistent activation distribution. The input sequence is converted into embeddings and added with positional encoding to feed the encoder, which produces an encoded representation of dimension $d_{model}=512$ after processing the input sequence.

### 2.2 Decoder

Similar to the encoder, the decoder is also composed of a six-layer stack. However, besides the two sub-layers of each encoder layer, it contains a third sub-layer for multi-head attention over the output of the encoder stack. Layer normalization [108] followed by residual connections [107] between sub-layers is applied in the decoder in the same way as in the encoder. During training, the target sequence is shifted by one position to enable the model to learn to predict the next word at each position in the sequence. Shifted target sequences are converted into the embeddings and added with positional encoding to generate input for the decoder. The self-attention sub-layer is masked to prevent the decoder from attending future tokens and to ensure that the predictions depend only on the known outputs. It is important to note that the number of layers in the encoder or decoder stack can be changed depending on the application and the available computational resources.

### 2.3 Attention

The self-attention sub-layer converts inputs into three vectors: query (**q**), key (**k**), and value (**v**). The dimensions of the vectors **q** and **k** are $d_{k}$, and the dimension of the vector **v** is $d_{v}$. For a particular key, a compatibility function of the query assigns a weight to each value. The weighted sum of the values is used to calculate the output. The objective of an attention function is to map a query and a set of key-value pairs with an output. The self-attention mechanism allows each token to pay attention to all other tokens in the sequence irrespective of distance, which helps capture long-range relationships. It makes the self-attention sub-layer different from standard neural network layers, which apply fixed-weight transformations to input tokens.

#### 2.3.1 Scaled dot-product attention

**Fig. 3:**
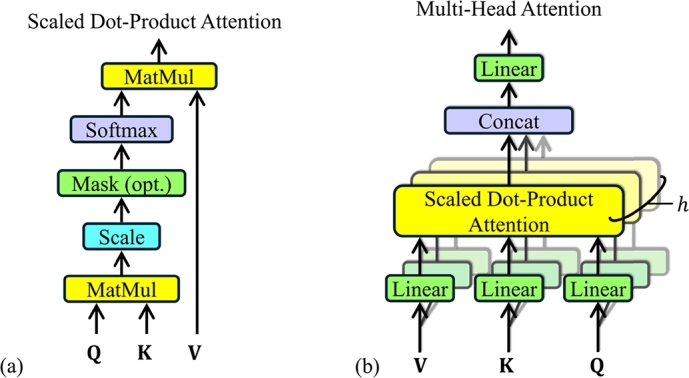
Schematic diagram of (a) Scaled dot-product attention and (b) Multi-head attention consisting several parallel attention layers [93].

The vectors obtained from different inputs are packed into three different matrices, **Q**, **K** and **V**, for query, key and value, respectively. A score is computed by dividing the dot products of **Q** and **K** by $\sqrt{d_{k}}$. This score is then applied to a softmax function to determine the weights of **V**, as shown in Fig. 3(a). Hence, the output of the attention function can be calculated using Equation (1):

(1) $$\text{Attention}(Q,K,V)=\text{softmax}\left(\frac{QK^{⊤}}{\sqrt{d_{k}}}\right)V$$

#### 2.3.2 Multi-head attention

A single-headed attention layer is unable to focus on multiple positions at the same time and pays attention only to its own location, causing poor model performance. To resolve this issue, the vanilla Transformer employed Multi-Head Attention. A schematic diagram of Multi-Head Attention is shown in Fig. 3 (b). It enables the model to focus on multiple positions in the input sequence by computing multiple attention functions in parallel. For linear projection of queries, keys, and values, the multi-head attention uses *h* heads, which reduces their dimensions. The vanilla Transformer used eight heads and reduced the dimensions of $d_{k}$ and $d_{v}$ ($d_{model}/h$) to 64. The computation of multi-head attention can be achieved by applying Equation (2) and Equation (3):

(2) $$\text{MultiHead}(Q,K,V)=\text{Concat}({\text{head}}_{1},\ldots ,{\text{head}}_{h})W^{O}$$

(3) $$\text{where }{\text{head}}_{i}=\text{Attention}(QW_{i}^{Q},KW_{i}^{K},VW_{i}^{V})$$

where the projections are parameter matrices: $W_{i}^{Q}\in R^{d_{model}\times d_{k}}$, $W_{i}^{K}\in R^{d_{model}\times d_{k}}$, $W_{i}^{V}\in R^{d_{model}\times d_{v}}$, and $W_{O}\in R^{hd_{v}\times d_{model}}$.

It is important to note that, unlike the self-attention layer in the encoder, the encoder-decoder attention layer in the decoder obtains **K** and **V** from the encoder stack's output, while **Q** is received from the previous sub-layer.

### 2.4 Position-wise feed-forward networks

As mentioned above, each layer of encoder and decoder contains a fully connected feed-forward network besides attention sub-layers. The purpose of this sub-layer is to improve the model representation by transforming the input vectors. To achieve this, it utilizes two linear transformations with a Rectified Linear Unit (ReLU) activation. For an input sequence $x\;=\;(x_{1},x_{2},\ldots ,x_{n})$, where *n* is the length of the sequence, this process can be represented by Equation (4).

(4) $$\text{FFN}(x)=\max(0,xW_{1}+b_{1})W_{2}+b_{2}$$

where $W_{1}$ and $W_{2}$ are the weight matrices of the two linear transformation layers, $b_{1}$ and $b_{2}$ are the biases for those layers. It is important to note that the non-linear activation ReLU is applied after the first linear transformation.

### 2.5 Embeddings and positional encoding

Artificial intelligence models cannot process real-world inputs and outputs, such as texts or images. Hence, to make it possible for the model to process these inputs and outputs, they must be represented numerically. This mathematical representation of inputs and outputs is known as embeddings. The Transformer utilizes embeddings to encode inputs and outputs into dense vectors that can be processed by the encoder and decoder.

Positional information is critical for sequential tasks. However, the self-attention sub-layer is incapable of capturing positional details. The vanilla Transformer solves this problem by adding positional encodings of the same dimension $d_{model}$ to the embeddings. Positional encodings are computed using Equation (5) and Equation (6).

(5) $${\text{PE}}_{\left(pos,2i\right)}=sin\left(\frac{pos}{10000^{\frac{2i}{d_{model}}}}\right)$$

(6) $${\text{PE}}_{\left(pos,2i+1\right)}=cos\left(\frac{pos}{10000^{\frac{2i}{d_{model}}}}\right)$$

where *pos* is the position in the sequence, *i* is the current dimension. The unique values generated by sine and cosine functions enable the model to differentiate between different positions within the sequence.

## 3 Transformer-based models for RNA structure prediction

Several factors hinder the accurate prediction of the RNA structures from the sequences, including complex folding dynamics, base pairing variability, sequence length variability, and lack of experimental data [109], [110], [111]. Another significant obstacle to developing prediction models is the lack of data for training and benchmarking [89]. The datasets and community-wide challenges most commonly used by recent structure prediction models to predict secondary or tertiary structures are listed in Table 1. Numerous Transformer-based models have recently been developed for RNA structure prediction, leveraging its ability to capture long-range dependencies and perform parallel calculations. This section reviews the Transformer-based models developed for prediction of the RNA structure.

**Table 1:** List of commonly used datasets and community-wide challenges by recent structure prediction models to predict secondary or tertiary structures.

Dataset/Challenges	RNA Sequence Length Range	Number of Sequences	Ref.
ArchiveII	28–2968	3975	[112]
bpRNA-1m	1–4381	102318	[13]
Protein Data Bank (PDB)	4–2902	669	[113]
Rfam	19–3932	30249	[114], [115]
RNAStralign	30–1851	37149	[116]
RNAcentral	201–244296	234669	[117]
RNAPuzzle	23–189	39	[118]
CASP15	30–720	12	[119]

### 3.1 Secondary structure

**Fig. 4:**
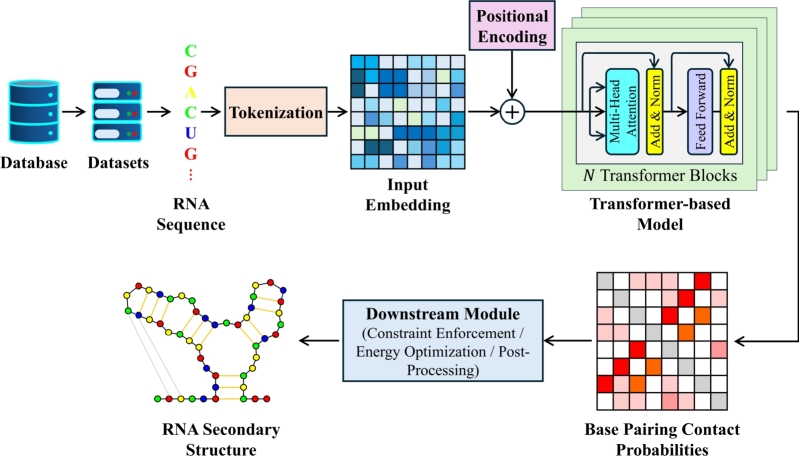
A schematic diagram representing the general workflow of Transformer-based secondary structure prediction models.

RNA secondary structure prediction can be simplified to identify which nucleotides in an RNA sequence are likely to form pairs with each other and which will remain unpaired [120], [121]. It also involves constraints, as some specific pairings cannot form, while some are more likely to occur. Leveraging the ability to perform sequential tasks efficiently [122], [123], researchers used the Transformer architecture to predict the RNA secondary structure. Fig. 4 illustrates a general workflow for secondary structure prediction using Transformer-based models. Table 2 summarizes the tools that predict the secondary structure of RNA using the Transformer architecture. F1 score is one of the commonly used metric to evaluate the performance of secondary structure prediction tools. The F1 score is defined as the harmonic mean of recall (REC) and precision (PR). REC of the model is the fraction of predicted base pairs in all native base pairs and can be calculated using Equation (7).

(7) $$REC=\frac{TP}{TP+FN}$$

**Table 2:** Summary of Transformer architecture-based models used for RNA secondary structure prediction.

Model	Model Input	Architecture	Model Output	Training Dataset	Validation Dataset	Test Dataset	Loss Function	F1 Score	Year	Ref.	Source Code URL
E2Efold	One-hot encoding + position embedding matrix	Transformer + CNN	Symmetric Matrix	RNAStralign	RNAStralign	ArchiveII and RNAStralign	Differentiable F1	0.821	2020	[124]	https://github.com/ml4bio/e2efold
ATTfold	One-hot encoding and Positional embeddings	Transformer + CNN	Base pairing scoring matrix	RNAStralign	RNAStralign	RNAStralign	Not specified	0.966	2020	[125]	https://github.com/YL-wang/ATTfold
LTPConstraint	Mapped embeddings	Bidirectional LSTM + Transformer + Unet	Pair scoring matrix	bpRNA, RNAStralign, 5SrRNA, tRNA, Rfam, and PDB	Rfam, 5SrRNA, tRNA, PDB	Rfam, 5SrRNA, tRNA, PDB	Weighed-logistic	0.9988	2022	[126]	https://github.com/jluF/LTPConstraint.git
GCNfold	One-hot encoding, position embeddings and probabilistic adjacency matrix	GCN + Transformer + CNN (U-Net)	Symmetric contact map	RNAStralign	RNAStralign	ArchiveII and RNAStralign	Binary cross-entropy	0.959	2023	[127]	https://github.com/EnbinYang/GCNfold
Knotfold	Embedding and positional encodings	Transformer	Base pairing probabilities	bpRNA-1m (TR0) and Rfam	bpRNA-1m (VL0) and Rfam	bpRNA-1m (TS0), Rfam and RfamNew (RfamNew)	Binary cross-entropy	0.667	2024	[128]	https://github.com/gongtiansu/KnotFold
RNAformer	Row-wise, column-wise embeddings and RoPE	Transformer + CNN	Binary pairing probability matrix	bpRNA-1m, ArchiveII, RNAStrAlign, RNA-Strand, and PDB	bpRNA-1m and PDB	bpRNA-1m and PDB	Binary cross-entropy	0.764	2024	[129]	https://github.com/automl/RNAformer
Wfold	RNA Sequence encoded into an image-like tensor	Transformer + CNN (U-net)	Probability-based contact matrix	RNAStralign and bpRNA-1m	Not specified	RNAStralign, ArchiveII, bpRNA-1m, bpRNA-new	Weighted binary cross-entropy	0.909	2024	[130]	https://github.com/EnjieYang/Wfold

PR of the model is the fraction of correctly predicted base pairs and can be calculated using Equation (8).

(8) $$PR=\frac{TP}{TP+FP}$$

where TP, FN, and FP are true positive, false negative and false positive. The F1 score of the model can be calculated by Equation (9).

(9) $$\text{F1 score}=\frac{2\times (REC\times PR)}{REC+PR}$$

The F1 score ranges from 0 to 1, where an F1 score of 1 indicates ideal performance of the model. The F1 scores reported in Table 2 are derived from the original publications of the methods and reflect the methodologies used by their respective authors. It is clear from Table 1 and Table 2 that every model is trained and tested on different datasets. Each dataset contains different RNA sequence length ranges, numbers of sequences, and RNA families. In the absence of any standard dataset, comparing the performance metrics of one model with another may lead to misinterpretation of the results. Therefore, datasets and the best F1 score for each tool are included in the table to highlight the absence of any standard dataset in RNA structure prediction and to encourage researchers to improve prediction accuracy and model robustness.

Chen et al. [124] proposed E2Efold, an end-to-end DL model for secondary structure prediction. They divided the E2Efold model into two parts. The first part is a Transformer-based network, Deep Score, that utilizes a stack of encoders to encode the sequence information and the global dependency between nucleotides. The network's input is one-hot embedding vectors of L×4 dimensions, where *L* is the length of the RNA sequence. The model also adds Bidirectional Encoder Representations from Transformers (BERT)-style [131] positional embeddings adapted to represent RNA sequences. 2D convolutional layers were used to generate pair-wise scores. The second part of the model is a multilayer post-processing network designed for constraint optimization using an unrolled algorithm. Despite achieving higher F1 scores than state-of-the-art methods at the time, Sato et al. [63] demonstrated that E2Efold performed poorly for unseen families with an F1 score of 0.0361. This inferior performance for unseen families was attributed to overfitting. Generally, base pairings in RNA lead to the formation of nested structural motifs such as hairpin loops, stems, and bulge loops [85]. However, pseudoknots base pairs are non-nested structures that overlap, making them difficult and computationally challenging for structure prediction models to predict [132]. ATTfold [125] utilizes the attention mechanism to predict secondary structures with and without pseudoknots. The architecture of ATTfold is divided into three parts: encoder, decoder, and constraint. The encoder combines one-hot encoded RNA sequences and positional encodings. The decoder then generates a base-pair scoring matrix using CNN. Finally, the constraint phase imposes hard constraints to obtain the final prediction matrix containing only 0 or 1 values, where 0 indicates that the two nucleotides do not form a pair and 1 implies that they form a pair. Its similarity to the E2Efold architecture suggests that ATTfold may also suffer from the generalization problem [90].

LTPConstraint [126] adopts a slightly different approach, as instead of the one-hot vector, the input of this model is a word vector with ten channels, which is converted from the sequence vector by an embedding layer. A global semantic extraction module is used to learn the representation of the word vector directly from the data. This extraction module contains a single-layer bidirectional LSTM network [133] and a Transformer Encoder with six layers of a 2-head self-attention module. The next stage is a generator of the pix2pix model, which employs skip connections like Unet. The generator generates a scoring matrix for each base pairing. Finally, the constraint layer of the third module creates an adjacency matrix. They also utilize transfer learning to reduce training cost and improve prediction accuracy. However, for longer sequences, the model still suffers from a high training cost and low prediction accuracy. With the goal of predicting an accurate secondary structure using fewer parameters, Yang et al. proposed the GCNfold [127]. GCNfold includes four components, three feature extractors, and a decoder. The Structure Extractor converts structural information into pairing probabilities using three Graph Convolutional Network (GCN) layers. Structure and Sequence Fusion concatenates the RNA and position vector as input to the three-layer Transformer Encoder with two multi-head attention layers to generate a feature map. It also utilizes absolute and relative position embeddings obtained from the E2Efold technique. The Long-distance Dependency Extractor has a UNet block composed of multiple convolutional and max-pooling layers to extract long-range pairwise relationships. Using 2D convolution, the decoder integrates the extractor outputs to create a contact map while retaining only one channel. The contact map is further processed to obtain a pairing matrix following the base pairing rules. GCNfold can process RNA sequences of up to 600 lengths. It also suffers from overfitting and struggles to generalize novel datasets.

To accurately predict the RNA secondary structures with pseudoknots, Gong et al. developed Knotfold using the Transformer architecture [128]. Initially, the embedding layer encodes the bases of the input RNA sequence of length *L* into learnable embeddings. The Transformer's encoder stack then processes these embeddings to generate an L×L basis pairing probability matrix, which is further transformed to compute a potential function. The structure with the lowest potential is determined using the minimum cost flow algorithm, and that structure is predicted as the RNA secondary structure. Furthermore, a loss function using binary cross-entropy over all possible base pairs is calculated to ensure prediction accuracy. Knotfold is also affected by poor generalization capabilities for new RNA families due to overfitting.

Franke et al. proposed a lean and scalable model by integrating attention mechanisms with CNNs for predicting RNA secondary structures with pseudoknots called RNAformer [129]. The model generates a 2D representation in its latent space that includes a row- and column-wise embedding for the input RNA sequence using rotary position embedding (ROPE) [134]. Based on the required scalability, the resulting latent representation is further handled by a stack of multiple RNAformer blocks. They have developed models with parameters ranging from 2M to 32M. Every block incorporates a row-wise and column-wise axial attention network, referred to as AxialAttentionNet, followed by a transition network TransitionConvNet. The transition network employs two convolutional layers with a SiLU activation function [135] to effectively capture local relationships between the nucleotides. After processing the 2D pair matrix, the stack of RNAformer blocks generates a binary pairing probability matrix of the secondary structure directly using a single linear layer. Since RNAformer requires large memory due to the two-dimensional representation in the latent space, the computational cost increases.

Wfold [130] combined a Transformer to capture long-range relationships, and a U-net, to learn local information, forming a W-shaped network. One of the advantages of this model is that it does not require the RNA sequences to be padded, as it can handle RNA sequences of any length. Wfold employs six layers of the Transformer as the encoder and U-net as the decoder. The input to the model is encoded into a one-hot vector represented by the L×4 binary matrix for an *L* nucleotide long RNA sequence. This matrix is then transformed into an image-like 16×L×L matrix considering all possible 16 base pairings. An additional channel is included to capture auxiliary real-world pairing information. Hence, the model accepts a 17×L×L tensor as input and generates an L×L symmetric matrix containing probability scores as output. The output probability matrix is further utilized to satisfy hard constraints to predict the secondary structure. The model is capable of predicting complex structures such as pseudoknots. However, the generalization capabilities of the model for the new datasets need to be verified.

### 3.2 RNA language models

RNA language models are being developed to solve the problem of scarcity of known RNA secondary and tertiary structures and annotated data. These RNA language models are designed to work upstream for different downstream tasks, including prediction of secondary and tertiary structures. Table 3 summarizes the RNA language models for RNA secondary structure prediction. Chen et al. proposed the RNA Foundation Model (RNA-FM) [136], which learns evolutionary information through self-supervised learning from 23 million unlabeled ncRNA sequences from the RNAcentral database [137]. RNA-FM employs a BERT [131] language model architecture consisting of twelve Transformer-based bidirectional encoder blocks to generate an L×640 embedding matrix for each RNA sequence of length *L*. It incorporates a dedicated task-specific module to fine-tune structure-related and function-related prediction performance. The Web server of RNA-FM provides access to the pre-trained model and embeddings.

**Table 3:** Summary of Transformer architecture-based RNA language models.

Model	Model Input	Architecture	Model Output	Training Dataset	Benchmark Dataset	Test Dataset	Loss Function	F1 Score	Year	Ref.	Source Code URL
RNA-FM	RNA sequences encoded as tokens	Transformer + ResNet	Embedding matrix	Pre-training: RNA Central Tuning: bpRNA-1m (TR0)	ArchiveII, and bpRNA-1m (VL0)	ArchiveII600, bpRNA-1m (TS0)	Cross-entropy	0.941	2022	[136]	https://github.com/ml4bio/RNA-FM
Uni-RNA	RNA sequences encoded as tokens and Rotary embedding	Transformer + ResNet	Binary 2D matrix	Pre-training: RNAcentral, nt (NCBI), Genome Warehouse (GWH) Fine tuning: RNAStralign, bpRNA-1m (TR0)	bpRNA-1m (VL0)	bpRNA-1m (TS0)	Cross-entropy	0.821	2023	[138]	Not available
RiNALMo	RNA sequences encoded as tokens and RoPE	Transformer + ResNet	Binary pairing probability matrix	RNAcentral, nt, Rfam, and Ensembl	Not Specified	bpRNA-1m (TS0)	Cross-entropy	0.75	2024	[139]	https://github.com/lbcb-sci/RiNALMo

Another model, UniRNA [138], utilized the BERT architecture [131] for pre-training one billion RNA sequences from different species, which allowed the model to learn effective representations of the complex dependencies of RNA structures. The model uses RoPE [134] to incorporate positional information into the encoder-only Transformer. Furthermore, to improve training efficiency and processing speed, Uni-RNA employs an IO-aware Flash Attention mechanism [140] instead of the multi-head attention. They proposed four versions of the model with 8, 12, 16, and 24 layers consisting of 8, 12, 16, and 20 attention heads, resulting in 25M, 85M, 169M, and 400M parameters, respectively. It was observed that although increasing the training data and model parameters improved model performance in downstream tasks, performance saturated when model parameters exceeded 400M. It is important to note that unlike RNA-FM, Uni-RNA is not publicly available.

In addition to RNA-FM and Uni-RNA, Penić et al. have recently proposed the largest RNA language model to date, the RiboNucleic Acid Language Model (RiNALMo). RiNALMo has 650 million parameters pre-trained on 36 million non-coding RNA sequences from databases including RNAcentral [137], Rfam [141], nt [142], and Ensembl [143]. It uses a BERT-style [131] encoder-only Transformer architecture comprising 33 Transformer blocks to learn local and global contextual relationships between nucleotides. The embedding layer of the encoder tokenizes each nucleotide of the input RNA sequence and transforms it into a 1280-dimension vector. Like Uni-RNA, RiNALMo employed RoPE [134] to capture relative and absolute positional information of tokens and IO-aware FlashAttention-2 [140] to improve the pre-training efficiency of such a large model. The multi-head attention of the encoder consists of 20 attention heads, and the SwiGLU activation function [144], which is a combination of the swish activation function and the gated linear unit (GLU), is used in the FFN with two linear layers. Pre-training of the RiNALMo is performed using masked language modeling (MLM) by corrupting unlabeled RNA sequences and then tasking the model to rebuild them. The length of the input model is restricted to a maximum of 1024 nucleotides owing to computational limitations. The beginning of each sequence is a classification token [CLS], and the end of the sequence is an end-of-sequence token [EOS]. Hence, any sequence longer than 1022 nucleotides is randomly clipped during each epoch. The trained model can be individually adjusted for various structural and functional downstream tasks, including RNA secondary structure prediction. The output embeddings of the model present a robust sequence representation that significantly enhances the model's performance. RiNALMo takes the RNA secondary structure prediction as a simple binary classification task with the binary cross-entropy loss and links a simple bottleneck ResNet [107] with the model to perform this task. The results demonstrated that the model was able to generalize well, even to RNA families that were not included during training, thus significantly compensating for the overfitting problem. After successfully making the model for predicting the RNA secondary structure, the authors have now started developing the model for predicting the RNA tertiary structure.

### 3.3 Tertiary structure

**Fig. 5:**
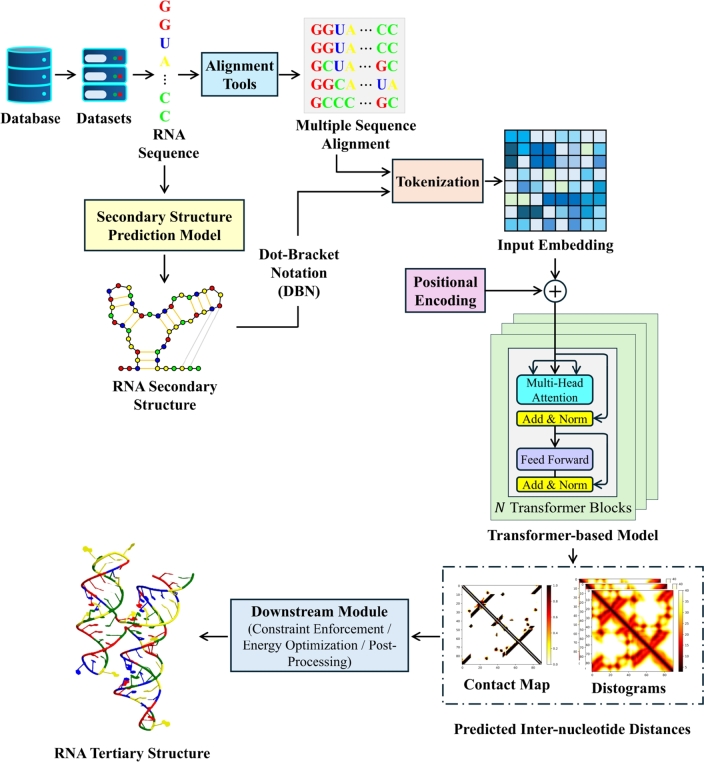
**A schematic diagram representing the general workflow of Transformer-based tertiary structure prediction models, illustrated using RNA-Puzzle Target 39**[150], [118]**.** The contact map, histograms, and tertiary structure predictions are performed using trRosettaRNA [16]. The tertiary structure is visualized using Jmol [17]. The structures used in this figure are for demonstration purpose only.

RNA tertiary structure prediction means predicting the spatial location of each atom in the RNA molecule for a given nucleic acid sequence [145]. Prediction models find it challenging to generate accurate folds even with experimental data and human assistance [146], [147]. Obtaining atomic resolution models of less than 2Å Root-Mean-Square Deviation (RMSD) is still difficult for current state-of-the-art methods [118], [148]. RNA tertiary structure prediction approaches can be classified as template-based and *de novo* methods. Template-based methods use homologous templates in the Protein Data Bank (PDB) [149] to predict the structural conformation. On the other hand, as the name suggests, *de novo* methods predict 3D structures by simulating the folding process from the start. Similarly to RNA secondary structure prediction, the use of the Transformer architecture in tertiary structure prediction has become increasingly popular. A general workflow of Transformer-based tertiary structure prediction models is illustrated in Fig. 5. Transformer-based RNA tertiary structure prediction methods are listed in Table 4. Generally, the average RMSD is one of the most commonly used metrics to evaluate the performance of tertiary structure prediction tools. Hence, the best average RMSD is given for each technique in Table 4. The average RMSDs reported in Table 4 are derived from the original publications of the methods and reflect the methodologies used by their respective authors.

**Table 4:** Summary of Transformer architecture-based models used for RNA tertiary structure prediction.

Model	Model Input	Architecture	Model Output	Training Dataset	Validation Dataset	Test Dataset	Loss Function	Average RMSD	Year	Ref.	Source Code URL
DeepFoldRNA	MSA, positional embeddings and PETfold2-SS	MSA Transformer + Sequence Transformer	Pairwise Distance and torsion angles maps	PDB and bpRNA-1m	Not specified	Rfam and RNA-Puzzles	Softmax cross-entropy	2.69Å	2022	[148]	https://github.com/robpearc/DeepFoldRNA
E2Efold-3D	MSA	RNA-FM + E2Eformer + Structure Module	Full atom model	PDB, BGSU RNA Representative Sets, RNAStralign, and bp-RNA-1m	Not specified	50 Curated RNAs and RNA puzzles	1D: Masked language modeling, 2D: Distance and secondary structure, 3D: Frame Aligned Point Error, and clash violation	2.938Å	2022	[151]	Not available
RoseTTAFoldNA	MSA	Special Euclidean Group Transformer	Full atom model	PDB	PDB	PDB	Frame Aligned Point Error	Not specified	2023	[152]	https://github.com/uw-ipd/RoseTTAFold2NA
trRosettaRNA	MSA and SPOT-RNA-secondary structure	RNAformer	Pairwise distance	PDB	Not specified	PDB (post-2017), RNA puzzles, and CASP15	Cross entropy	8.5Å	2023	[16]	https://yanglab.nankai.edu.cn/trRosettaRNA/.
DRfold	One-hot, RNAfold-secondary structure, and PETfold-secondary structure	Transformer	Full atom model	PDB	Not specified	40 RNA Rest set and CASP15	Frame Aligned Point Error and inter-N atom distance	14.45Å	2023	[153]	https://zhanggroup.org/DRfold
RhoFold+	Embeddings and MSA	RNA-FM + Rhoformer + Structure Module	Full atom model	RNAcentral, Rfam, PDB, RNAStralign and bpRNA-1m	Tenfold cross-validation	RNA puzzles, CASP15	1D: Masked language modeling, 2D: Distance and secondary structure, 3D: Frame Aligned Point Error, 3D Secondary Structure Constraint, and clash violation loss	5.47Å	2024	[154]	https://github.com/ml4bio/RhoFold

To predict RNA tertiary structure, Pearce et al. proposed DeepFoldRNA, which uses the Transformer architecture with gradient-based folding simulations, specifically, limited-memory Broyden–Fletcher–Goldfarb–Shano (L-BFGS) minimization simulations [148]. The model performs the structure prediction task in three steps: input feature generation, spatial restraint prediction, and L-BFGS folding simulations. The input to the DeepFoldRNA model is a nucleic acid sequence in FASTA (Fast-All) format. Pairwise reliability scores based on generated MSA are computed using PETfold [155] for secondary structure prediction. In addition to the reliability scores, a pair representation is also included as a feature for input embeddings. These features are processed through 48 MSA Transformer blocks followed by 4 Sequence Transformer blocks to learn the spatial dependencies between each pair of positions. For refining purposes, the whole procedure is repeated four times. The distance and orientation restraints are derived from a linear projection of the last pair representation, and for the prediction of the backbone torsion angles, a linear projection of the sequence representation is used.

An end-to-end approach to predict tertiary structures is introduced in the E2Efold-3D technique [151]. The PDB and BGSU RNA Representative sets are used to train the model, and the self-distillation dataset is derived from RNAStralign [116] and bp-RNA-1m [13]. Initially, MSA and pairwise residue features are extracted from the Rfam and RNACentral databases through the Infernal [156] and rMSA [53] protocol using the RNA sequence as a query. The MSA is then encoded into a one-hot vector, which is transformed into an embedding after combining it with positional encoding. These features are merged with the features obtained from the 12-layer RNA-FM model [136] and fed into a 4-layer E2Eformer module to capture the sequence representation and base interactions. E2Eformer has row- and column-wise gated attention layers to generate an attention matrix. After refining the output sequence and pair representation, E2Eformer feeds it to the structure module. Finally, the 8-layer structure module employs geometry-aware attention operation and invariant point attention (IPA) to determine 3D positions and predict RNA tertiary structures. Baek et al. modified RoseTTAFold [157], originally designed to predict the protein structure, to RoseTTAFoldNA [152] for the prediction of RNA tertiary structure. RoseTTAFold's three-track architecture pays attention to three different representations. The 1D, 2D, and 3D tracks of RoseTTAFoldNA simultaneously update sequence, pairwise relationships, and structural information, respectively, with the help of the SE(3) (special Euclidean group in three dimensions) Transformers. Complexes with more than 1,000 total amino acids and nucleotides were excluded only for the validation set due to the memory limitations of the graphics processing unit (GPU).

Wang et al. developed trRosettaRNA with the aim of optimizing the global RMSD in RNA 3D structure predictions [16]. The first step of the process is to prepare the MSAs and secondary structures as input to the model. The MSAs are developed using rMSA with different databases (NCBI's nt, Rfam, and RNAcentral) and Infernal with the RNAcentral database. While SPOT-RNA [61] is used to generate a probability matrix from the query sequence. These representations are processed by a Transformer-based model (RNAformer) to predict the 1D and 2D geometries. It is important to clarify here that Franke has also developed another method for secondary structure prediction, called RNAformer, which we discussed in the previous section. However, the same name can sometimes be confusing. The RNAformer of trRosettaRNA consists of 48 blocks of Transformers and is cycled four times for complete prediction. Finally, full-atom structure models are generated using energy minimization with learning potentials and physics-based energy terms in Rosetta. However, trRosettaRNA struggles to generalize unseen structures and suffers from overfitting problems.

DRfold [153] develops an end-to-end learning model that optimizes the predicted conformations by geometry-based potentials. For effective training and faithful structure reconstruction, it integrates geometric modules with coarse-grained modeling where the nucleotides are represented by the phosphate (P), ribose (C4′), and glycosidic (N) atoms in the coarse-grained model. DRfold converts the input RNA sequence into a 5-D one-hot encoded vector that contains 4 types of nucleotides and an unknown state (N) representing modified or degenerated nucleotides. DRfold employs predictions for secondary structures from RNAfold [158] and PETfold [155]. It also adds positional encodings and finally generates 64-dimensional sequence and pair embeddings. The design of the Transformer block is inspired by the Evoformer in AlphaFold2 [159]. It also has some similarities with DeepfoldRNA as both contain 48 Transformer blocks. Each Transformer block uses 8 heads and 8 channels. The Transformer blocks process the representations using advanced geometric modules, including triangle multiplicative updates and triangle self-attention, and generate rotation matrices and translation vectors. The rotation matrices and translation vectors are utilized to determine the atomic coordinates of the RNA structure. Another set of Transformer blocks learns RNA inter-nucleotide geometry information in parallel, using pair representations. A composite potential is generated by adding predicted frame vectors and geometric restraints for gradient-based RNA structure reconstruction. Finally, the conformation with the lowest energy is used to reconstruct the atomic-level structure using the coarse-grained models. However, due to insufficient training data, the model could not achieve the expected performance.

A recently developed fully automated end-to-end pipeline for RNA 3D structure prediction called RhoFold+ [154] addresses the issue of data scarcity by employing the RNA-FM language model [136], which we discussed in section 3.2. The RhoFold+ pipeline can be divided into three main parts: RNA-FM, Rhoformer module, and Structure module. Initially, RNA-FM with 12 encoder blocks converts the input RNA sequences into embeddings by capturing evolutionary and structural information. Additionally, MSA features were generated using rMSA and Infernal. Next in the pipeline comes the Rhoformer module, which uses gated self-attention layers to learn evolutionary information, as well as update pairwise sequence embeddings and MSA representations. From sequence and pair representations, the structure module predicts the RNA tertiary structure. It also adopts a recycling technique to optimize structural output based on feedback from earlier predictions. Local frame coordinates and torsion angles for critical atoms in the RNA backbone are refined using a geometry-aware attention mechanism and an invariant point attention (IPA) module. Despite significant improvements in prediction accuracy, Rhofold+ experiences difficulty in predicting complex RNA structures such as pseudoknots, particularly RNA sequences with 500 or more nucleotides.

**Fig. 6:**
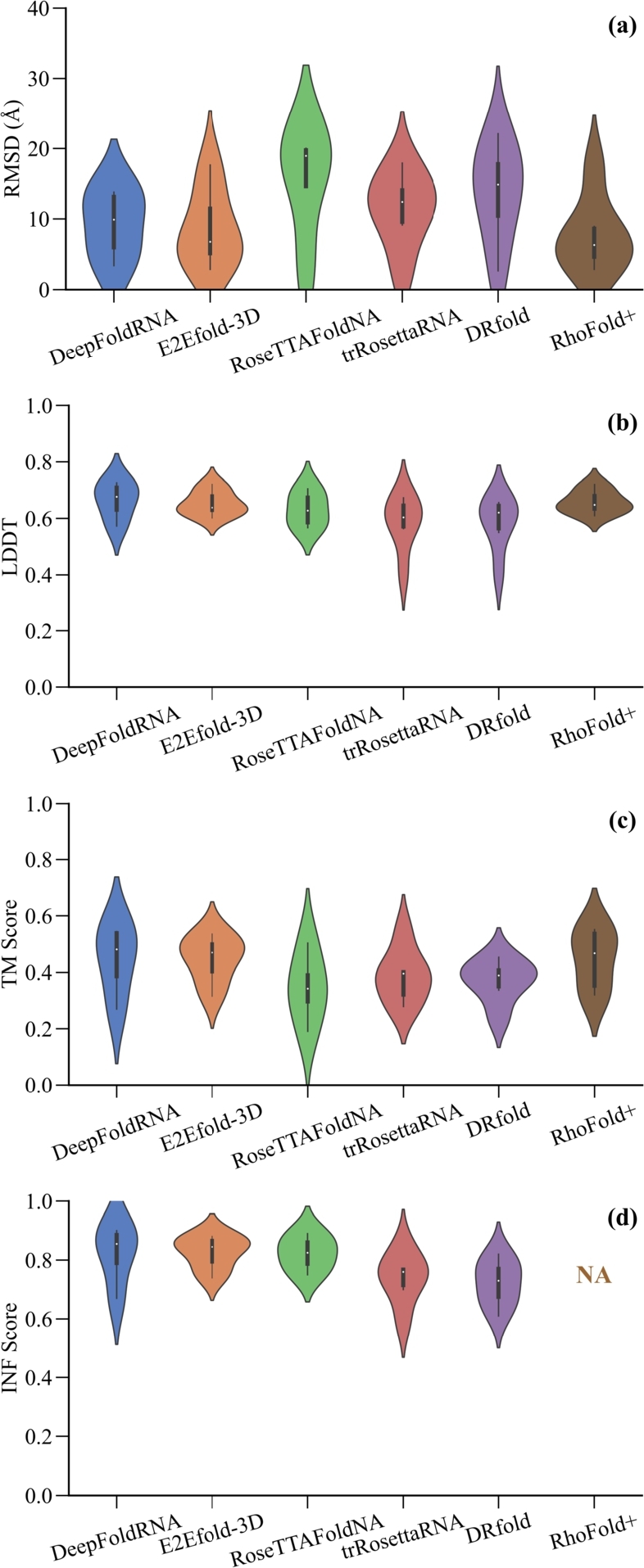
**A comparison of Transformer-based models' performance in CASP15 natural RNA targets: (a) Root-Mean-Square Deviation (RMSD) (b) Local Distance Difference Test (LDDT) (c) Template Modeling Score (TM-Score) (d) Interaction Network Fidelity (INF) Score.** Data is obtained from the official CASP15 website [119], [160] and the original publication of the RhoFold+ model [154]. NA indicates data not available or non participation.

It is important to note that community-wide blind test structure prediction competitions such as RNA-Puzzles and Critical Assessment of Structure Prediction (CASP) are helping to advance computational biology by improving predictive models for RNA. In these competitions, researchers/research groups voluntarily participate and submit predicted RNA structures based on sequence information, which are compared with experimentally determined structures to assess the quality of the model. Most of the models in Table 4 participated in CASP15, where they were evaluated on key metrics such as RMSD, Local Distance Difference Test (LDDT), Template Modeling Score (TM-score), and Interaction Network Fidelity (INF) score. The LDDT provides an estimate of the local quality of the predicted structure by measuring the local distance difference among all atoms in a predicted structure. The TM-score is used to evaluate the structural similarity between the predicted and the reference RNA structures. The INF score measures the accuracy of predicted base-pairing interactions within the predicted RNA structure and shows how well the predicted interactions match known interactions in the reference structure. A lower RMSD denotes better accuracy, whereas higher LDDT, TM-score, and INF score indicate better model quality. Table 5 shows the RMSD values achieved on six CAPS15 natural RNA targets by the Transformer-based tertiary structure prediction models listed in Table 4 to provide an objective analysis. Furthermore, a comparison of Transformer-based models' performance in CASP15 natural RNA targets based on RMSD, LDDT, TM-score, and INF score is shown in Fig. 6. The data in Table 5 and Fig. 6 are sourced from the official CASP15 website and the original publication of the RhoFold+ model.

**Table 5:** Comparison of performance based on Root-Mean-Square Deviation (RMSD) obtained by the Transformer-based RNA tertiary structure prediction models on CASP15 natural RNA targets. Data is obtained from the official CASP15 website [119], [160] and the original publication of the RhoFold+ model [154]. NA indicates data not available or non participation.

Model	Group name registered in CASP15	RMSD (Å)						
		R1107	R1108	R1116	R1117	R1149	R1156	Average
DeepFoldRNA	DF_RNA	NA	NA	13.80	3.40	6.88	12.90	9.25
E2Efold-3D	AIchemy_RNA	5.92	4.91	12.69	2.87	7.66	17.65	8.62
RoseTTAFoldNA	BAKER	NA	NA	18.26	3.73	19.63	20.03	15.41
trRosettaRNA	Yang-Server	17.9	29.13	10.88	2.70	13.93	14.09	11.44
DRfold	rDP	18.10	9.59	12.99	2.67	16.75	22.10	13.70
RhoFold+	NA	5.01	4.54	8.92	2.87	7.66	17.43	7.74

## 4 Discussion

The works analyzed in this review show that Transformer-based RNA structure prediction models have significantly improved prediction accuracy for both secondary and tertiary structures. Generally, in RNA structure prediction tasks, model performance is evaluated using the F1 score and RMSD for secondary and tertiary structures, respectively. However, every model uses different datasets for training and testing and employs different computational resources for processing, which makes comparisons between models challenging.

Several reasons can be attributed to the higher performance of the Transformer architecture in RNA structure prediction tasks. Transformers are adept at handling sequential input, so they can easily extract features from RNA sequences [161], [162], [163]. Moreover, the nucleotides in the RNA molecule interact to form complex structures. Transformers can efficiently capture long-range dependencies between nucleotides because of the ability of the attention mechanism to focus on global relationships. Another key factor is the capability of Transformers to perform parallel computations, which enables them to simultaneously analyze patterns across the entire input RNA sequence, facilitating the capture of long-range dependencies. Transformer-based models, particularly large language models, can also be pre-trained on extensive datasets, which helps them learn features effectively. These pre-trained models can be used for various downstream tasks, including RNA structure prediction.

Despite exhibiting promising results, there is still much room for improvement. The lack of experimentally confirmed, high-resolution (≤ 2 Å) data poses a significant challenge in achieving higher prediction accuracy [164], [89]. Millions of RNA sequences are added to the RNACentral database every year. However, the pace of adding high-resolution RNA tertiary structures to the PDB database has been relatively slow. In 2024, 35,400,760 RNA sequences were deposited in the latest release of RNACentral, Release 24. In contrast, as of February 2025, 8,420 known RNA 3D structures had been deposited in the PDB. Only 937 structures were deposited in the PDB in 2024. Hence, in addition to reducing the accuracy of structure prediction, the lack of reliable data results in poor generalization. Models can be standardized using family-fold cross-validation to mitigate the generalization problem for unseen datasets [165], [166].

Besides the slow deposition rate of RNA structures, the lack of widely accepted quality standards leads to the deposition of substandard RNA data [167]. Generally, base pairs available in public archives are unreliable because they may be incomplete, incorrect, or missing critical formal details [168]. The use of low-quality base pairing data adversely impacts the model's performance. Consequently, extensive benchmarking of public records is necessary, as well as ensuring that they are regularly updated [167].

Most of the transformer-based tertiary structure prediction models we have discussed use MSAs as input. However, producing high-quality RNA alignments is complex and often requires expert interventions [169]. That is why the number of alignments in Rfam [114], the database that collects RNA alignments, is inadequate to effectively train DL models. Furthermore, global biases in the Rfam alignments introduce additional complications [167].

Generally, for longer RNA sequences, prediction accuracy decreases while computational cost increases [170]. Furthermore, Transformers need larger datasets to learn features, and as a result, the number of parameters and computational cost escalates. Therefore, there is a need to immediately focus on improving performance and optimizing computational cost. Another complication with the RNA structure is RNA folding. It is difficult to determine whether the RNA is completely folded into its functional structure or exists in an intermediate state [171]. To address this problem, information related to intermediate states should be included in the training dataset.

## 5 Conclusion

Structural analysis of RNA sequences using computational techniques has facilitated a deeper understanding of RNA functions and the development of RNA-based therapeutics. Transformers have become increasingly popular in RNA structure prediction tasks owing to their advantages, such as capturing long-range dependencies and the ability to perform parallel calculations. This article provides an overview of Transformer-based techniques for predicting secondary and tertiary structures from RNA sequences. So far, attempts have been made to modify the vanilla Transformer architecture to solve the RNA structure prediction problem, but have achieved only partial success. Additionally, the lack of high resolution RNA structures in the PDB remains a serious challenge, causing the structure prediction models to suffer from overfitting and failure to generalize well. Pre-trained RNA language models have demonstrated improved generalization ability for RNA secondary structure prediction by capturing hidden but important structural information from unlabeled RNA sequences. However, considerable progress is still needed in the field of prediction of tertiary structures. Furthermore, an important future direction should be the development of highly effective and efficient structure prediction models with high performance and low resource costs. Finally, we hope that this review provides a valuable resource for researchers to understand the progress made in Transformer-based RNA structure prediction methods and devise more efficient techniques.

## Funding

This work was supported by 10.13039/501100000923The Australian Research Council [DP210101875 to Kuldip K. Paliwal].

## CRediT authorship contribution statement

**Mayank Chaturvedi:** Writing – review & editing, Writing – original draft, Visualization, Validation, Software, Methodology, Investigation, Formal analysis, Data curation, Conceptualization. **Mahmood A. Rashid:** Writing – review & editing, Validation. **Kuldip K. Paliwal:** Writing – review & editing, Supervision, Resources, Project administration, Funding acquisition.

## Declaration of Competing Interest

The authors declare that they have no known competing financial interests or personal relationships that could have appeared to influence the work reported in this paper.

## References

[1] He L., Hannon G.J. (2004). MicroRNAs: small RNAs with a big role in gene regulation. Nat Rev Genet.

[2] Morris K.V., Mattick J.S. (2014). The rise of regulatory RNA. Nat Rev Genet.

[3] Tinoco I., Bustamante C. (1999). How RNA folds. J Mol Biol.

[4] Spitale R.C., Flynn R.A., Torre E.A., Kool E.T., Chang H.Y. (2014). RNA structural analysis by evolving SHAPE chemistry. Wiley Interdiscip Rev: RNA.

[5] Deng J., Fang X., Huang L., Li S., Xu L., Ye K. (2023). RNA structure determination: from 2D to 3D. Fundam Res.

[6] Jones C.P., Ferré-D'Amaré A.R. (2022). Crystal structure of the severe acute respiratory syndrome coronavirus 2 (SARS-CoV-2) frameshifting pseudoknot. RNA.

[7] Gumna J., Antczak M., Adamiak R.W., Bujnicki J.M., Chen S.-J., Ding F. (2022). Computational pipeline for reference-free comparative analysis of RNA 3D structures applied to SARS-CoV-2 UTR models. Int J Mol Sci.

[8] Minchin S., Lodge J. (2019). Understanding biochemistry: structure and function of nucleic acids. Essays Biochem.

[9] Westhof E., Fritsch V. (2000). RNA folding: beyond Watson–Crick pairs. Structure.

[10] Xu D., Landon T., Greenbaum N.L., Fenley M.O. (2007). The electrostatic characteristics of G· U wobble base pairs. Nucleic Acids Res.

[11] Do C.B., Woods D.A., Batzoglou S. (2006). CONTRAfold: RNA secondary structure prediction without physics-based models. Bioinformatics.

[12] Andronescu M., Bereg V., Hoos H.H., Condon A. (2008). RNA STRAND: the RNA secondary structure and statistical analysis database. BMC Bioinform.

[13] Danaee P., Rouches M., Wiley M., Deng D., Huang L., Hendrix D. (2018). bpRNA: large-scale automated annotation and analysis of RNA secondary structure. Nucleic Acids Res.

[14] Olson W.K., Li S., Kaukonen T., Colasanti A.V., Xin Y., Lu X.-J. (2019). Effects of noncanonical base pairing on RNA folding: structural context and spatial arrangements of G·A pairs. Biochemistry.

[15] Kerpedjiev P., Hammer S., Hofacker I.L. (2015). Forna (force-directed RNA): simple and effective online RNA secondary structure diagrams. Bioinformatics.

[16] Wang W., Feng C., Han R., Wang Z., Ye L., Du Z. (2023). trRosettaRNA: automated prediction of RNA 3D structure with transformer network. Nat Commun.

[17] Jmol: an open-source Java viewer for chemical structures in 3D. http://www.jmol.org/.

[18] Jaeger L., Chworos A. (2006). The architectonics of programmable RNA and DNA nanostructures. Curr Opin Struct Biol.

[19] Grabow W.W., Jaeger L. (2014). RNA self-assembly and RNA nanotechnology. Acc Chem Res.

[20] Copley S.D., Smith E., Morowitz H.J. (2007). The origin of the RNA world: co-evolution of genes and metabolism. Bioorg Chem.

[21] Shoemaker S.C., Ando N. (2018). X-rays in the cryo-electron microscopy era: structural biology's dynamic future. Biochemistry.

[22] Wilkinson K.A., Merino E.J., Weeks K.M. (2006). Selective 2′-hydroxyl acylation analyzed by primer extension (SHAPE): quantitative RNA structure analysis at single nucleotide resolution. Nat Protoc.

[23] Smola M.J., Rice G.M., Busan S., Siegfried N.A., Weeks K.M. (2015). Selective 2′-hydroxyl acylation analyzed by primer extension and mutational profiling (SHAPE-MaP) for direct, versatile and accurate RNA structure analysis. Nat Protoc.

[24] Wang X.-W., Liu C.-X., Chen L.-L., Zhang Q.C. (2021). RNA structure probing uncovers RNA structure-dependent biological functions. Nat Chem Biol.

[25] Kubota M., Tran C., Spitale R.C. (2015). Progress and challenges for chemical probing of RNA structure inside living cells. Nat Chem Biol.

[26] Mitchell D., Assmann S.M., Bevilacqua P.C. (2019). Probing RNA structure *in vivo*. Curr Opin Struct Biol.

[27] Ehresmann C., Baudin F., Mougel M., Romby P., Ebel J.-P., Ehresmann B. (1987). Probing the structure of RNAs in solution. Nucleic Acids Res.

[28] Kotar A., Foley H.N., Baughman K.M., Keane S.C. (2020). Advanced approaches for elucidating structures of large RNAs using NMR spectroscopy and complementary methods. Methods.

[29] Lietzke S.E., Barnes C.L., Kundrot C.E. (1995). Crystallization and structure determination of RNA. Curr Opin Struct Biol.

[30] Wirecki T.K., Merdas K., Bernat A., Boniecki M.J., Bujnicki J.M., Stefaniak F. (2020). RNAProbe: a web server for normalization and analysis of RNA structure probing data. Nucleic Acids Res.

[31] Gumna J., Zok T., Figurski K., Pachulska-Wieczorek K., Szachniuk M. (2020). RNAthor – fast, accurate normalization, visualization and statistical analysis of RNA probing data resolved by capillary electrophoresis. PLoS ONE.

[32] Nussinov R., Pieczenik G., Griggs J.R., Kleitman D.J. (1978). Algorithms for loop matchings. SIAM J Appl Math.

[33] Zuker M., Stiegler P. (1981). Optimal computer folding of large RNA sequences using thermodynamics and auxiliary information. Nucleic Acids Res.

[34] Ding Y., Lawrence C. (2003). A statistical sampling algorithm for RNA secondary structure prediction. Nucleic Acids Res.

[35] Sato K., Hamada M., Asai K., Mituyama T. (2009). CentroidFold: a web server for RNA secondary structure prediction. Nucleic Acids Res.

[36] Hofacker I.L. (2003). Vienna RNA secondary structure server. Nucleic Acids Res.

[37] Huang L., Zhang H., Deng D., Zhao K., Liu K., Hendrix D.A. (2019). LinearFold: linear-time approximate RNA folding by 5'-to-3'dynamic programming and beam search. Bioinformatics.

[38] Zuker M. (2003). Mfold web server for nucleic acid folding and hybridization prediction. Nucleic Acids Res.

[39] Cao S., Chen S.-J. (2005). Predicting RNA folding thermodynamics with a reduced chain representation model. RNA.

[40] Parisien M., Major F. (2008). The MC-Fold and MC-Sym pipeline infers RNA structure from sequence data. Nature.

[41] Zadeh J., Steenberg C., Bois J., Wolfe B., Pierce M., Khan A. (2011). NUPACK: analysis and design of nucleic acid systems. J Comput Chem.

[42] Xia T., SantaLucia J., Burkard M.E., Kierzek R., Schroeder S.J., Jiao X. (1998). Thermodynamic parameters for an expanded nearest-neighbor model for formation of RNA duplexes with Watson-Crick base pairs. Biochemistry.

[43] Markham N., Zuker M. (2008). UNAfold: software for nucleic acid folding and hybridization. Methods Mol Biol.

[44] Reuter J.S., Mathews D.H. (2010). RNAstructure: software for RNA secondary structure prediction and analysis. BMC Bioinform.

[45] Hochsmann M., Toller T., Giegerich R., Kurtz S. (2003). Computational systems bioinformatics. CSB2003. Proceedings of the 2003 IEEE bioinformatics conference.

[46] Bernhart S.H., Hofacker I.L., Will S., Gruber A.R., Stadler P.F. (2008). RNAalifold: improved consensus structure prediction for RNA alignments. BMC Bioinform.

[47] Yao Z., Weinberg Z. (2020). CMfinder–a covariance model based RNA motif finding algorithm. Bioinformatics.

[48] Touzet H., Perriquet O. (2004). CARNAC: folding families of related RNAs. Nucleic Acids Res.

[49] Fu Y., Sharma G., Mathews D.H. (2014). Dynalign II: common secondary structure prediction for RNA homologs with domain insertions. Nucleic Acids Res.

[50] Rivas E., Clements J., Eddy S.R. (2017). A statistical test for conserved RNA structure shows lack of evidence for structure in lncRNAs. Nat Methods.

[51] Rivas E. (2020). RNA structure prediction using positive and negative evolutionary information. PLoS Comput Biol.

[52] Zhang C., Zheng W., Mortuza S., Li Y., Zhang Y. (2020). DeepMSA: constructing deep multiple sequence alignment to improve contact prediction and fold-recognition for distant-homology proteins. Bioinformatics.

[53] Zhang C., Zhang Y., Pyle A.M. (2023). rMSA: a sequence search and alignment algorithm to improve RNA structure modeling. J Mol Biol.

[54] Rivas E., Lang R., Eddy S.R. (2012). A range of complex probabilistic models for RNA secondary structure prediction that includes the nearest-neighbor model and more. RNA.

[55] Akiyama M., Sato K., Sakakibara Y. (2018). A max-margin training of RNA secondary structure prediction integrated with the thermodynamic model. J Bioinform Comput Biol.

[56] Akiyama M., Sakakibara Y., Sato K. (2022). Direct inference of base-pairing probabilities with neural networks improves prediction of RNA secondary structures with pseudoknots. Genes.

[57] Lu W., Tang Y., Wu H., Huang H., Fu Q., Qiu J. (2019). Predicting RNA secondary structure via adaptive deep recurrent neural networks with energy-based filter. BMC Bioinform.

[58] Willmott D., Murrugarra D., Ye Q. (2020). Improving RNA secondary structure prediction via state inference with deep recurrent neural networks. Comput Math Biophys.

[59] Lu W., Cao Y., Wu H., Ding Y., Song Z., Zhang Y. (2021). Research on RNA secondary structure predicting via bidirectional recurrent neural network. BMC Bioinform.

[60] Zhang H., Zhang C., Zhang B., Liu Y. (2019). A new method of RNA secondary structure prediction based on convolutional neural network and dynamic programming. Front Genet.

[61] Singh J., Hanson J., Paliwal K., Zhou Y. (2019). RNA secondary structure prediction using an ensemble of two-dimensional deep neural networks and transfer learning. Nat Commun.

[62] Fu L., Cao Y., Wu J., Peng Q., Nie Q., Xie X. (2022). UFold: fast and accurate RNA secondary structure prediction with deep learning. Nucleic Acids Res.

[63] Sato K., Akiyama M., Sakakibara Y. (2021). RNA secondary structure prediction using deep learning with thermodynamic integration. Nat Commun.

[64] Das S., Tariq A., Santos T., Kantareddy S.S., Banerjee I. (2023). Recurrent neural networks (RNNs): architectures, training tricks, and introduction to influential research. Mach Learn Brain Disord.

[65] Hochreiter S. (1998). The vanishing gradient problem during learning recurrent neural nets and problem solutions. Int J Uncertain Fuzziness Knowl-Based Syst.

[66] Kim H., Nam H., Jung W., Lee J. (2017). 2017 IEEE international symposium on performance analysis of systems and software (ISPASS).

[67] Rashid M.A., Paliwal K.K. (2024). Distance-based contact maps prediction for RNA bases using deep neural networks and single sequence features. Int J Bioinform Res Appl.

[68] Rashid M.A., Paliwal K.K. (2023). 2023 IEEE Asia-Pacific conference on computer science and data engineering (CSDE).

[69] Kipf T.N., Welling M. (2016). Semi-supervised classification with graph convolutional networks. https://arxiv.org/abs/1609.02907.

[70] Xu K., Hu W., Leskovec J., Jegelka S. (2018). How powerful are graph neural networks?. https://arxiv.org/abs/1810.00826.

[71] Oono K., Suzuki T. (2019). Graph neural networks exponentially lose expressive power for node classification. https://arxiv.org/abs/1905.10947.

[72] Nt H., Maehara T. (2019). Revisiting graph neural networks: all we have is low-pass filters. https://arxiv.org/abs/1905.09550.

[73] Nithin C., Kmiecik S., Błaszczyk R., Nowicka J., Tuszyńska I. (2024). Comparative analysis of RNA 3D structure prediction methods: towards enhanced modeling of RNA–ligand interactions. Nucleic Acids Res.

[74] Pasquali S., Derreumaux P. (2010). HiRE-RNA: a high resolution coarse-grained energy model for RNA. J Phys Chem B.

[75] Krokhotin A., Houlihan K., Dokholyan N.V. (2015). IFoldRNA v2: folding RNA with constraints. Bioinformatics.

[76] Li J., Chen S.-J. (2023). RNAJp: enhanced RNA 3D structure predictions with non-canonical interactions and global topology sampling. Nucleic Acids Res.

[77] Popenda M., Szachniuk M., Antczak M., Purzycka K.J., Lukasiak P., Bartol N. (2012). Automated 3D structure composition for large RNAs. Nucleic Acids Res.

[78] Boniecki M.J., Lach G., Dawson W.K., Tomala K., Lukasz P., Soltysinski T. (2016). SimRNA: a coarse-grained method for RNA folding simulations and 3D structure prediction. Nucleic Acids Res.

[79] Watkins A.M., Rangan R., Das R. (2020). FARFAR2: improved *De Novo* Rosetta prediction of complex global RNA folds. Structure.

[80] Zhang Y., Wang J., Xiao Y. (2020). 3dRNA: building RNA 3D structure with improved template library. Comput Struct Biotechnol J.

[81] Zhou L., Wang X., Yu S., Tan Y.-L., Tan Z.-J. (2022). FebRNA: an automated fragment-ensemble-based model for building RNA 3D structures. Biophys J.

[82] Li J., Zhu W., Wang J., Li W., Gong S., Zhang J. (2018). RNA3DCNN: local and global quality assessments of RNA 3D structures using 3D deep convolutional neural networks. PLoS Comput Biol.

[83] Zhang S., Liu Y., Xie L. (2022). Physics-aware graph neural network for accurate RNA 3D structure prediction. https://arxiv.org/abs/2210.16392.

[84] Congzhou M.S., Wang J., Dokholyan N.V. (2024). Predicting 3D RNA structure from the nucleotide sequence using Euclidean neural networks. Biophys J.

[85] Chaturvedi M., Rashid M.A., Paliwal K.K. (2025). RNA structure prediction using deep learning—a comprehensive review. Comput Biol Med.

[86] Dawson W.K., Bujnicki J.M. (2016). Computational modeling of RNA 3D structures and interactions. Curr Opin Struct Biol.

[87] Zhao Q., Zhao Z., Fan X., Yuan Z., Mao Q., Yao Y. (2021). Review of machine learning methods for RNA secondary structure prediction. PLoS Comput Biol.

[88] Sato K., Hamada M. (2023). Recent trends in RNA informatics: a review of machine learning and deep learning for RNA secondary structure prediction and RNA drug discovery. Brief Bioinform.

[89] Justyna M., Antczak M., Szachniuk M. (2023). Machine learning for RNA 2D structure prediction benchmarked on experimental data. Brief Bioinform.

[90] Wu K.E., Zou J.Y., Chang H. (2023). Machine learning modeling of RNA structures: methods, challenges and future perspectives. Brief Bioinform.

[91] Wang X., Yu S., Lou E., Tan Y.-L., Tan Z.-J. (2023). RNA 3D structure prediction: progress and perspective. Molecules.

[92] Budnik M., Wawrzyniak J., Grala Ł., Kadziński M., Szóstak N. (2024). Deep dive into RNA: a systematic literature review on RNA structure prediction using machine learning methods. Artif Intell Rev.

[93] Vaswani A., Shazeer N., Parmar N., Uszkoreit J., Jones L., Gomez A.N. (2017). Attention is all you need. Adv Neural Inf Process Syst.

[94] Khan S., Naseer M., Hayat M., Zamir S.W., Khan F.S., Shah M. (2022). Transformers in vision: a survey. ACM Comput Surv.

[95] Chen X., Xie S., He K. (2021). 2021 IEEE/CVF international conference on computer vision (ICCV).

[96] Huang Z., Ben Y., Luo G., Cheng P., Yu G., Fu B. (2021). Shuffle transformer: rethinking spatial shuffle for vision transformer. https://arxiv.org/abs/2106.03650.

[97] Islam R., Maktoomi M.H., Ren H., Arigong B. (2021). Spectrum aggregation dual-band real-time RF/microwave analog signal processing from microstrip line high-frequency Hilbert transformer. IEEE Trans Microw Theory Tech.

[98] Şahinuç F., Koç A. (2022). Fractional Fourier transform meets transformer encoder. IEEE Signal Process Lett.

[99] Parisotto E., Song F., Rae J., Pascanu R., Gulcehre C., Jayakumar S., III H.D., Singh A. (2020). Proceedings of the 37th international conference on machine learning.

[100] Hu S., Shen L., Zhang Y., Chen Y., Tao D. (2024). On transforming reinforcement learning with transformers: the development trajectory. IEEE Trans Pattern Anal Mach Intell.

[101] Meng L., Goodwin M., Yazidi A., Engelstad P. (2024). Proceedings of the 2024 8th international conference on digital signal processing.

[102] Melo L.C., Chaudhuri K., Jegelka S., Song L., Szepesvari C., Niu G., Sabato S. (2022). Proceedings of the 39th international conference on machine learning.

[103] Yu C., Xu Y., Cao J., Zhang Y., Jin Y., Zhu M. (2024). 2024 IEEE international conference on metaverse computing, networking, and applications (MetaCom).

[104] Lin J., Guo X., Zhu Y., Mitchell S., Altman E., Shun J. (2024). Proceedings of the 5th ACM international conference on AI in finance.

[105] Yang X., Zhang C., Sun Y., Pang K., Jing L., Wa S. (2023). FinChain-BERT: a high-accuracy automatic fraud detection model based on NLP methods for financial scenarios. Information.

[106] Tian Y., Liu G. (2024). Spatial-temporal-aware graph transformer for transaction fraud detection. IEEE Trans Ind Inform.

[107] He K., Zhang X., Ren S., Sun J. (2016). Proceedings of the IEEE conference on computer vision and pattern recognition (CVPR).

[108] Ba J.L., Kiros J.R., Hinton G.E. (2016). Layer normalization. https://arxiv.org/abs/1607.06450.

[109] Xue Y., Gracia B., Herschlag D., Russell R., Al-Hashimi H.M. (2016). Visualizing the formation of an RNA folding intermediate through a fast highly modular secondary structure switch. Nat Commun.

[110] Kretsch R.C., Andersen E.S., Bujnicki J.M., Chiu W., Das R., Luo B. (2023). RNA target highlights in CASP15: evaluation of predicted models by structure providers. Proteins, Struct Funct Bioinform.

[111] Zhang S., Li J., Chen S.-J. (2024). Machine learning in RNA structure prediction: advances and challenges. Biophys J.

[112] Sloma M.F., Mathews D.H. (2016). Exact calculation of loop formation probability identifies folding motifs in RNA secondary structures. RNA.

[113] Berman H.M., Battistuz T., Bhat T.N., Bluhm W.F., Bourne P.E., Burkhardt K. (2002). The protein data bank. Acta Crystallogr, Sect D, Biol Crystallogr.

[114] Griffiths-Jones S., Bateman A., Marshall M., Khanna A., Eddy S.R. (2003). Rfam: an RNA family database. Nucleic Acids Res.

[115] Ontiveros-Palacios N., Cooke E., Nawrocki E.P., Triebel S., Marz M., Rivas E. (2025). Rfam 15: RNA families database in 2025. Nucleic Acids Res.

[116] Tan Z., Fu Y., Sharma G., Mathews D.H. (2017). TurboFold II: RNA structural alignment and secondary structure prediction informed by multiple homologs. Nucleic Acids Res.

[117] T. RNAcentral Consortium (2019). RNAcentral: a hub of information for non-coding RNA sequences. Nucleic Acids Res.

[118] Bu F., Adam Y., Adamiak R.W., Antczak M., de Aquino B.R.H., Badepally N.G. (2025). RNA-puzzles round V: blind predictions of 23 RNA structures. Nat Methods.

[119] Das R., Kretsch R.C., Simpkin A.J., Mulvaney T., Pham P., Rangan R. (2023). Assessment of three-dimensional RNA structure prediction in CASP15. Proteins, Struct Funct Bioinform.

[120] Lorenz R., Wolfinger M.T., Tanzer A., Hofacker I.L. (2016). Predicting RNA secondary structures from sequence and probing data. Methods.

[121] Bindewald E., Shapiro B.A. (2006). RNA secondary structure prediction from sequence alignments using a network of k-nearest neighbor classifiers. RNA.

[122] Lin T., Wang Y., Liu X., Qiu X. (2022). A survey of transformers. AI Open.

[123] Cao K., Zhang T., Huang J. (2024). Advanced hybrid LSTM-transformer architecture for real-time multi-task prediction in engineering systems. Sci Rep.

[124] Chen X., Li Y., Umarov R., Gao X., Song L. (2020). RNA secondary structure prediction by learning unrolled algorithms. https://arxiv.org/abs/2002.05810.

[125] Wang Y., Liu Y., Wang S., Liu Z., Gao Y., Zhang H. (2020). ATTfold: RNA secondary structure prediction with pseudoknots based on attention mechanism. Front Genet.

[126] Fei Y., Zhang H., Wang Y., Liu Z., Liu Y. (2022). LTPConstraint: a transfer learning based end-to-end method for RNA secondary structure prediction. BMC Bioinform.

[127] Yang E., Zhang H., Zang Z., Zhou Z., Wang S., Liu Z. (2023). GCNfold: a novel lightweight model with valid extractors for RNA secondary structure prediction. Comput Biol Med.

[128] Gong T., Ju F., Bu D. (2024). Accurate prediction of RNA secondary structure including pseudoknots through solving minimum-cost flow with learned potentials. Commun Biol.

[129] Franke J.K., Runge F., Koeksal R., Backofen R., Hutter F. (2024). RNAformer: a simple yet effective deep learning model for RNA secondary structure prediction. bioRxiv.

[130] Yuan Y., Yang E., Zhang R. (2024). Wfold: a new method for predicting RNA secondary structure with deep learning. Comput Biol Med.

[131] Devlin J., Chang M., Lee K., Toutanova K., Burstein J., Doran C., Solorio T. (2019). Proceedings of NAACL-HLT 2019.

[132] Hajdin C.E., Bellaousov S., Huggins W., Leonard C.W., Mathews D.H., Weeks K.M. (2013). Accurate SHAPE-directed RNA secondary structure modeling, including pseudoknots. Proc Natl Acad Sci USA.

[133] Graves A., Schmidhuber J. (2005). Framewise phoneme classification with bidirectional LSTM and other neural network architectures. Neural Netw.

[134] Su J., Ahmed M., Lu Y., Pan S., Bo W., Liu Y. (2024). RoFormer: Enhanced transformer with rotary position embedding. Neurocomputing.

[135] Elfwing S., Uchibe E., Doya K. (2018). Sigmoid-weighted linear units for neural network function approximation in reinforcement learning. Neural Netw.

[136] Chen J., Hu Z., Sun S., Tan Q., Wang Y., Yu Q. (2022). Interpretable RNA foundation model from unannotated data for highly accurate RNA structure and function predictions. https://arxiv.org/abs/2204.00300.

[137] (2021). RNAcentral 2021: secondary structure integration, improved sequence search and new member databases. Nucleic Acids Res.

[138] Wang X., Gu R., Chen Z., Li Y., Ji X., Ke G. (2023). UNI-RNA: universal pre-trained models revolutionize RNA research. bioRxiv.

[139] Penić R.J., Vlašić T., Huber R.G., Wan Y., Šikić M. (2024). RiNALMo: general-purpose RNA language models can generalize well on structure prediction tasks. https://arxiv.org/abs/2403.00043.

[140] Dao T. (2023). Flashattention-2: faster attention with better parallelism and work partitioning. https://arxiv.org/abs/2307.08691.

[141] Kalvari I., Nawrocki E.P., Ontiveros-Palacios N., Argasinska J., Lamkiewicz K., Marz M. (2021). Rfam 14: expanded coverage of metagenomic, viral and microRNA families. Nucleic Acids Res.

[142] Sayers E.W., Bolton E.E., Brister J.R., Canese K., Chan J., Comeau D.C. (2023). Database resources of the national center for biotechnology information in 2023. Nucleic Acids Res.

[143] Martin F.J., Amode M.R., Aneja A., Austine-Orimoloye O., Azov A.G., Barnes I. (2023). Ensembl 2023. Nucleic Acids Res.

[144] Shazeer N. (2020). GLU variants improve transformer. https://arxiv.org/abs/2002.05202.

[145] Liu Z., Yang Y., Li D., Lv X., Chen X., Dai Q. (2022). Prediction of the RNA tertiary structure based on a random sampling strategy and parallel mechanism. Front Genet.

[146] Miao Z., Adamiak R.W., Antczak M., Batey R.T., Becka A.J., Biesiada M. (2017). RNA-puzzles round III: 3D RNA structure prediction of five riboswitches and one ribozyme. RNA.

[147] Miao Z., Adamiak R.W., Antczak M., Boniecki M.J., Bujnicki J., Chen S.-J. (2020). RNA-puzzles round IV: 3D structure predictions of four ribozymes and two aptamers. RNA.

[148] Pearce R., Omenn G.S., Zhang Y. (2022). *De Novo* RNA tertiary structure prediction at atomic resolution using geometric potentials from deep learning. bioRxiv.

[149] Burley S.K., Bhikadiya C., Bi C., Bittrich S., Chen L., Crichlow G.V. (2021). RCSB protein data bank: powerful new tools for exploring 3D structures of biological macromolecules for basic and applied research and education in fundamental biology, biomedicine, biotechnology, bioengineering and energy sciences. Nucleic Acids Res.

[150] Das N.K., Hollmann N.M., Vogt J., Sevdalis S.E., Banna H.A., Ojha M. (2023). Crystal structure of a highly conserved enteroviral 5' cloverleaf RNA replication element. Nat Commun.

[151] Shen T., Hu Z., Peng Z., Chen J., Xiong P., Hong L. (2022). E2Efold-3D: end-to-end deep learning method for accurate *de novo* RNA 3D structure prediction. https://arxiv.org/abs/2207.01586.

[152] Baek M., McHugh R., Anishchenko I., Jiang H., Baker D., DiMaio F. (2024). Accurate prediction of protein–nucleic acid complexes using RoseTTAFoldNA. Nat Methods.

[153] Li Y., Zhang C., Feng C., Pearce R., Lydia Freddolino P., Zhang Y. (2023). Integrating end-to-end learning with deep geometrical potentials for ab initio RNA structure prediction. Nat Commun.

[154] Shen T., Hu Z., Sun S., Liu D., Wong F., Wang J. (2024). Accurate RNA 3D structure prediction using a language model-based deep learning approach. Nat Methods.

[155] Seemann S.E., Menzel P., Backofen R., Gorodkin J. (2011). The PETfold and PETcofold web servers for intra- and intermolecular structures of multiple RNA sequences. Nucleic Acids Res.

[156] Nawrocki E.P., Eddy S.R. (2013). Infernal 1.1: 100-fold faster RNA homology searches. Bioinformatics.

[157] Baek M., DiMaio F., Anishchenko I., Dauparas J., Ovchinnikov S., Lee G.R. (2021). Accurate prediction of protein structures and interactions using a three-track neural network. Science.

[158] Lorenz R., Bernhart S.H., Höner zu Siederdissen C., Tafer H., Flamm C., Stadler P.F. (2011). ViennaRNA package 2.0. Algorithms Mol Biol.

[159] Jumper J., Evans R., Pritzel A., Green T., Figurnov M., Ronneberger O. (2021). Highly accurate protein structure prediction with alphafold. Nature.

[160] Prediction Center Protein Structure (2022). Casp15 - 15th critical assessment of techniques for protein structure prediction. https://predictioncenter.org/casp15/.

[161] Zhao H., Zhu B., Jiang T., Cui Z., Wu H. (2023). International conference on intelligent computing.

[162] Zeng A., Chen M., Zhang L., Xu Q. (2023). Proceedings of the AAAI conference on artificial intelligence.

[163] Sadad T., Aurangzeb R.A., Imran, Safran M., Alfarhood S., Kim J. (2023). Classification of highly divergent viruses from DNA/RNA sequence using transformer-based models. Biomedicines.

[164] Townshend R.J., Eismann S., Watkins A.M., Rangan R., Karelina M., Das R. (2021). Geometric deep learning of RNA structure. Science.

[165] Szikszai M., Wise M., Datta A., Ward M., Mathews D.H. (2022). Deep learning models for RNA secondary structure prediction (probably) do not generalize across families. Bioinformatics.

[166] de Lajarte A.A., Martin des Taillades Y.J., Kalicki C., Fuchs Wightman F., Aruda J., Salazar D. (2024). Diverse database and machine learning model to narrow the generalization gap in RNA structure prediction. bioRxiv.

[167] Schneider B., Sweeney B.A., Bateman A., Cerny J., Zok T., Szachniuk M. (2023). When will RNA get its AlphaFold moment?. Nucleic Acids Res.

[168] Baptista R.P., Kissinger J.C. (2019). Is reliance on an inaccurate genome sequence sabotaging your experiments?. PLoS Pathog.

[169] Wilm A., Higgins D.G., Notredame C. (2008). R-coffee: a method for multiple alignment of non-coding RNA. Nucleic Acids Res.

[170] Zhao Q., Mao Q., Zhao Z., Yuan W., He Q., Sun Q. (2023). RNA independent fragment partition method based on deep learning for RNA secondary structure prediction. Sci Rep.

[171] Wan Y., Mitchell D., Russell R. (2009).

